# Characterization of the mitochondrial genome of *Spodoptera exempta* (Lepidoptera: Noctuidae) from South Africa

**DOI:** 10.1080/23802359.2020.1869619

**Published:** 2021-02-08

**Authors:** Zaiyuan Li, Wenkai Wang, Yutao Xiao, Lei Zhang

**Affiliations:** a Forewarning and Management of Agricultural and Forestry Pests, Hubei Engineering Technology Center, Institute of Entomological Science, College of Agriculture, Yangtze University, Jingzhou, China; b Agricultural Genomics Institute at Shenzhen, Chinese Academy of Agricultural Sciences, Shenzhen, China

**Keywords:** *Spodoptera exempta*, mitogenome, phylogenetic analysis, Lepidoptera

## Abstract

The African armyworm, *Spodoptera exempta*, is an episodic migratory crop pest with an expanding distribution worldwide. This is the first report of the circular mitochondrial genome of *S. exempta*, with a length of 15,457 bp and an A + T content of 81.7%. It encoded a common set of 37 genes, including 13 protein-coding genes (PCGs), 22 tRNA genes, and two rRNA genes, and contained a putative control region of 379 bp (94.7% in A + T proportion). The maximum-likelihood phylogenetic tree based on the complete mitogenome demonstrated that five species belonging to the *Spodoptera* genus formed one clade, in which *S. exempta* was the most isolated branch, followed by *Spodoptera exigua*. This data will contribute for the identification and phylogenetic analyses of *S. exempta*, providing useful information for its comprehensive control.

The African armyworm, *Spodoptera exempta* (Walker, 1856) (Lepidoptera: Noctuidae), is an episodic crop pest and the most potentially serious hazard among migratory pests in Africa (Brown and Swaine, 1966; Pringle 1980; Redman et al. 2010). Mass oviposition of *S*. *exempta* results in the initiation of larval outbreaks, which can cause severe damage to rangeland grasses and crops, including maize, millet, rice, wheat, among others (Parker and Gatehouse 1985a, 1985b; Xu et al. 2020). To date, *S*. *exempta* has been widely distributed worldwide, including Africa, Asia, North America, and Oceania (Gunn and Gatehouse 1985; Haggis 1986). In the last two years, the fall armyworm, *Spodoptera frugiperda*, has invaded India and most Southeast Asian countries, as well as China (Zhang et al. 2020). As a species from the same genus, *S*. *exempta* has gained public attention again given its potential damaging effect for East Asian countries, including China, Japan, Korea. The species identification and correct differentiation of *S*. *exempta* are very important for accurate monitoring and early control. In the present study, the complete mitochondrial genome sequence of *S*. *exempta* was determined.

The *S*. *exempta* specimen used in this study was obtained from an inbred strain established from eggs collected in 2014 near Greytown, South Africa (29°03′ S, 30°36′ E). The genomic DNA of a male moth was extracted using the Qiagen Genomic DNA kit (Cat. no.13323, Qiagen) followed by purity assessment with a NanoDrop One UV-Vis spectrophotometer (Thermo Fisher Scientific). The voucher specimen’s genomic DNA (AGIS-SE-ZA-2014) deposited at the Agricultural Genomics Institute in Shenzhen, China. A total of 0.5 μg of genomic DNA was used to construct a 350-bp insert library, which was then sequenced using the 150-bp paired-end mode in an Illumina NovaSeq 6000 system (San Diego, CA, USA). The mitogenome was assembled using the software NOVOPlasty v2.5.6 (Dierckxsens et al. 2017), and its annotation was conducted using MITOS2 (http://mitos2.bioinf.uni-leipzig.de/index.py) (Bernt et al. 2013).

The complete mitogenome of *S*. *exempta* was a typical circular DNA molecule with 15,457 bp. The length was the longest compared with other four published *Spodoptera* species, which ranged from 15,365 to 15,388 bp (Wan et al. 2013; Wu et al. 2013; Seo et al. 2019). The mitogenome comprised 13 protein-coding genes (PCGs), 22 transfer RNA (tRNAs) genes, two ribosomal RNA genes, and one predicted control region. All five *Spodoptera* species, including *S*. *exempta*, shared the same gene order and possessed the ancestral gene order with *trnM-trnI-trnQ* of lepidopteran mitogenomes (Cao et al. 2012). The A + T content of the mitogenome was 81.7%, which was similar to that of other *Spodoptera* species (80.9 ∼ 81.3%) and fell within the range of the A + T content for other Lepidoptera species (Kim et al. 2006; Salvato et al. 2008). The start codon of all PCGs was ATN (where N represents A, T, or G), except for *cox1* that begun with CGA. Most PCGs were terminated with TAA or TAG as the stop codon, with the exception of *cox2* and *nad4* that had the incomplete stop codon T, which could produce the functional stop codon TAA by the addition of 3’A residues in polyadenylation processes (Ojala et al. 1981). The 379-bp control region of *S*. *exempta* was located between *srRNA* and *trnM*, which are known to be involved in the initiation of transcription and/or replication in other insects (Zhang and Hewitt 1997). All tRNAs harbored typical predicted secondary cloverleaf structures, except *trnS1* and *trnY*.

In order to determine the evolutionary relationship of *S. exempta*, a total of 25 complete mitogenomes (including 23 species in Heteroneura and 2 outgroup species from Exoporia) were used in phylogenetic analysis. 25 complete mitogenome sequences were multiple-aligned using MAFFT v7.455 (Katoh and Standley 2013). The phylogenetic tree was constructed based on the maximum likelihood method using RAxML-NG v0.9.0 (Kozlov et al. 2019) with the best-fit model (GTR + F+R4) estimated by IQ-TREE v1.6.10 with the parameter ‘-m MF’ (Nguyen et al. 2015). The bootstrap replicates were 1,000 and the tree was visualized with FigTree v1.4.4 (http://tree.bio.ed.ac.uk/software/figtree/). The phylogenetic tree confirmed that *S*. *exempta* belongs to the *Spodoptera* genus with a high nodal supporting value, presenting the species relationships ([([*S*. *littoralis* + *S*. *litura*] + *S*. *frugiperda*) + *S*. *exigua*] + *S*. *exempta*) (Figure 1). Different families of Lepidoptera and subfamilies of Noctuidae form one clade. The present data will contribute for the identification and phylogenetic assessment of *S*. *exempta*, and may provide useful information for the comprehensive control of hazardous crop pests.

**Figure 1.**
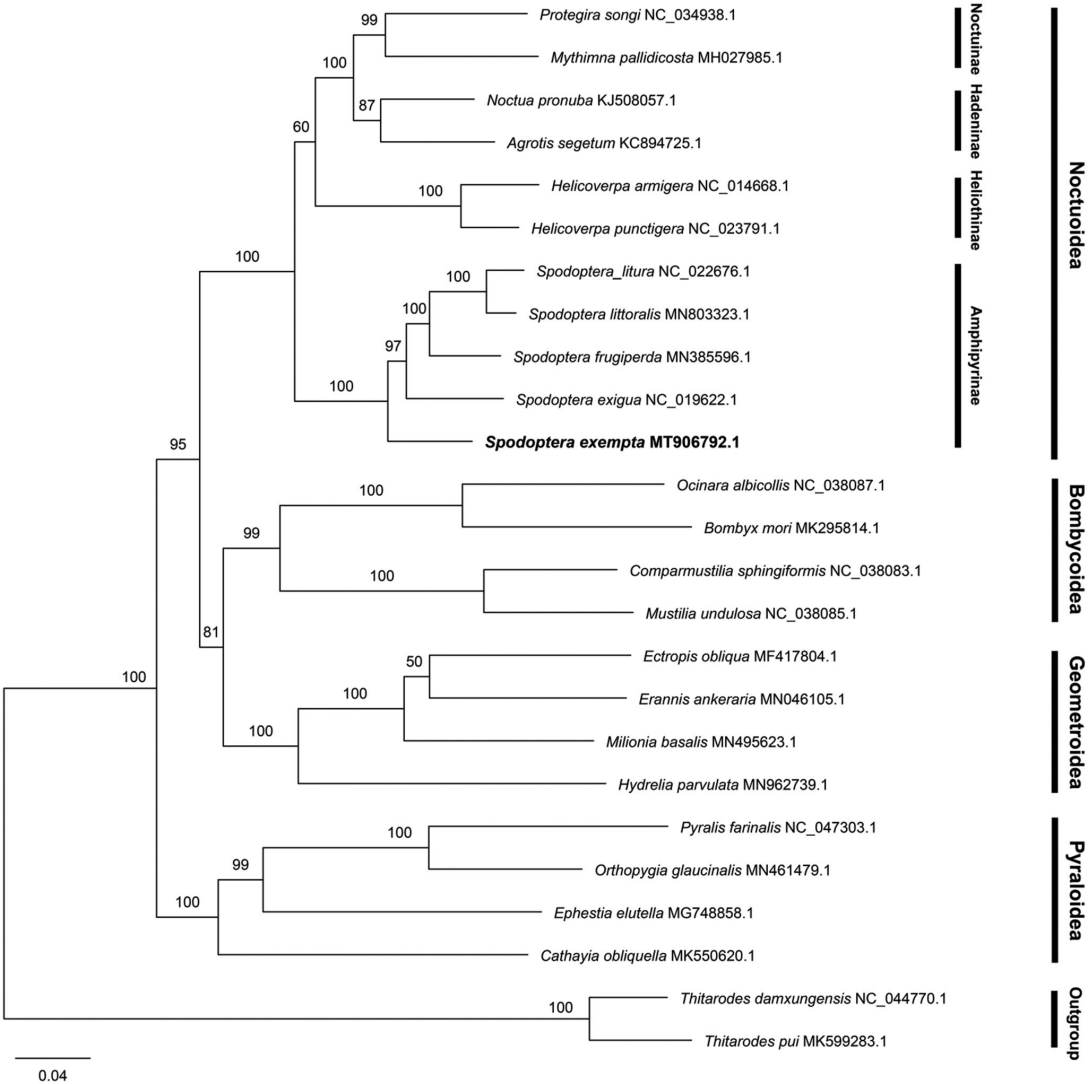
Phylogenetic tree of 25 species based on complete mitogenome. *Thitarodes damxungensis* and *Thitarodes pui* were used as outgroups. The numbers above the branches indicate the bootstrap values of the maximum likelihood tree.

## Data Availability

Mitogenome data supporting this study are openly available in GenBank at: https://www.ncbi.nlm.nih.gov/nuccore/MT906792. Associated BioProject, BioSample and SRA accession numbers are PRJNA678819, SAMN16814351, and SRR13083393, respectively.
